# Genes *WHEAT FRIZZY PANICLE* and *SHAM RAMIFICATION 2* independently regulate differentiation of floral meristems in wheat

**DOI:** 10.1186/s12870-017-1191-3

**Published:** 2017-12-28

**Authors:** Oxana B. Dobrovolskaya, Yumiko Amagai, Karina I. Popova, Alina E. Dresvyannikova, Petr Martinek, Alexander A. Krasnikov, Nobuyoshi Watanabe

**Affiliations:** 1 Institute of Cytology and Genetics, SB RAS, Lavrenvieva ave. 10, Novosibirsk, 630090 Russia; 2 Novosibirsk State University, Pirogova, 2, Novosibirsk, 630090 Russia; 3 College of Agriculture, Ibaraki University, Ibaraki, Japan; 4 Agrotest Fyto, Ltd., Kroměříž, Czech Republic; 5 Central Siberian Botanical Garden SB RAS, Novosibirsk, Russia

**Keywords:** *Frizzypanicle*, *Sham ramification 2*, Spike branching, False-true ramification, Grasses, Wheat, Spike, Meristem, Inflorescence development

## Abstract

**Background:**

Inflorescences of wheat species, spikes, are characteristically unbranched and bear one sessile spikelet at a spike rachis node. Development of supernumerary spikelets (SSs) at rachis nodes or on the extended rachillas is abnormal. Various wheat morphotypes with altered spike morphology, associated with the development of SSs, present an important genetic resource for studies on genetic regulation of wheat inflorescence development.

**Results:**

Here we characterized diploid and tetraploid wheat lines of various non-standard spike morphotypes, which allowed for identification of a new mutant allele of the *WHEAT FRIZZY PANICLE* (*WFZP*) gene that determines spike branching in diploid wheat *Ttiticum monococcum* L. Moreover, we found that the development of SSs and spike branching in wheat *T. durum* Desf. was a result of a *wfzp-A/TtBH-A1* mutation that originated from spontaneous hybridization with *T. turgidum* convar. *сompositum* (L.f.) Filat. Detailed characterization of the false-true ramification phenotype controlled by the recessive *sham ramification 2* (*shr2*) gene in tetraploid wheat *T. turgidum* L. allowed us to suggest putative functions of the *SHR2* gene that may be involved in the regulation of spikelet meristem fate and in specification of floret meristems. The results of a gene interaction test suggested that genes *WFZP* and *SHR2* function independently in different processes during spikelet development, whereas another spike ramification gene(s) interact(s) with *SHR2* and share(s) common functions.

**Conclusions:**

SS mutants represent an important genetic tool for research on the development of the wheat spikelet and for identification of genes that control meristem activities. Further studies on different non-standard SS morphotypes and wheat lines with altered spike morphology will allow researchers to identify new genes that control meristem identity and determinacy, to elucidate the interaction between the genes, and to understand how these genes, acting in concert, regulate the development of the wheat spike.

**Electronic supplementary material:**

The online version of this article (10.1186/s12870-017-1191-3) contains supplementary material, which is available to authorized users.

## Background

The flower is the most important reproductive organ of angiosperms, and is directly related to the processes of pollination, fertilization, development of the embryo, and the formation of seeds and fruits. Cereal flowers, florets, develop on a specialized short branch called a spikelet. This is the basal unit of the cereal inflorescence and is a characteristic of all modern grasses, except for only one early-diverged lineage [1]. Spikelets may develop either directly on the inflorescence axis or from primary or secondary inflorescence axillary meristems. The pattern of branching and specification of spikelet meristems determines the architecture of the cereal inflorescence. Wheat inflorescence, the spike, is characteristically unbranched, and sessile spikelets are directly attached to the spike axis (rachis) at rachis nodes. Spikelets consist of two glumes and 2—5 fertile florets arranged along an indeterminate axis, the spikelet rachilla. The number of spikelets per a rachis node is a key taxonomic characteristic of the Triticeae tribe [2, 3]. In all wheats, including diploid *Triticum monococcum* L. (2*n =* 2*×* = 14, genome formula AA), tetraploid *T. durum* Desf. (2*n =* 2*× =* 28, BBAA), and hexaploid *T. aestivum* L. (2*n* = 2*×* = 42, BBAADD) species, normally one spikelet develops at a spikelet node and formation of supernumerary spikelets (SSes) or branch-like structures is considered an abnormality and observed rarely. The exception is tetraploid wheat *Triticum turgidum* L. Forms of *T. turgidum* with spike branching have been known for 2000 years; Pliny the Elder mentioned them under the name *ramosum* and *centigranum* (23—79 AD) [4]. Because of the tendency to form branching spikes, this tetraploid wheat has been variously called e.g. “Miracle wheat”, “Mummy”, “Egyptian”, “Jerusalem wheat”, “Seven-headed” [4, 5]. Now *T. turgidum* forms with branching spikes are classified as *T. turgidum* convar. *compositum* (L.f) A. Filat. All *T. turgidum* branching forms have a common spike phenotype: additional spikelets develop on branches and directly at rachis nodes in the lower one-third of the spike; this spike phenotype is often referred to as the *turgidum* type of branching. In another tetraploid species, *T. durum* Desf. (BBAA), forms with spike branching are rare [4, 6]. Coffman [7] has described spontaneous mutants with SSes that have been found in the Mindum durum wheat variety. These mutants are characterized by the occurrence of one or two additional sessile spikelets at several rachis nodes in the lower part of the spike; the phenotype is different from the *turgidum* spike branching. Spontaneous mutant forms with spike branching have been found by M.M. Jakubziner in two *T. durum* cultivars: Akmolinka 5 and Hordeiforme [8]. V.F. Dorofeev has found *T. durum* forms with a spike branching phenotype similar to the *turgidum* type of branching in local mixed populations of tetraploid (*T. durum*, *T. turgidum*) and hexaploid (*T. aestivum*) wheat species grown in Transcaucasia [8]. Forms with spike branching and sessile SSes have also been found in tetraploid *T. dicoccum* (Schrank) Schuebl. and occasionally in *T. polonicum* L. species [6].

In diploid wheat species, spontaneous mutants with spike branching have not been reported [9], whereas an induced mutant that is characterized by the development of SSes at rachis nodes and on extended spikelet rachilla (on branches) was obtained by Dr. Yamashita in the second half of the last century [10].

In hexaploid bread wheat *T. aestivum* L., SS forms, including spike branching forms, are rare. Percival [6] described forms with sessile SSes in bread wheat, whose spike phenotypes are similar to that of the spontaneous mutant of the Mindum durum wheat variety [7]. Forms with the SS phenotype have been obtained as a result of intra- and interspecific hybridization, treatment with radioactive isotopes, or chemical mutagens, and spontaneous mutagenesis [11–14]. Martinek and Bednař [15, 16] have recorded several bread wheat morphotypes with SSes: multirow spike (MRS, a large number of spikelets emerge from each rachis node in the lower one-third of the spike); horizontal spikelets (HS), syn. ‘tetrastichon sessile spikelets’(two or three spikelets are in a horizontal position at a spike rachis node); vertical or pared spikelets, (VS, two spikelets arise vertically at a spike rachis node), this phenotype also called banana or tween spikelets; and genuine branching (GB), or ramified spike (RS), in addition to the sessile SSes, lateral branches bearing spikelets are formed in the basal part of the spike. This group of SS lines also includes the triple spikelet phenotype of Tibetan triple-spikelet wheat [14], as a type of HS. The GB spike phenotype of hexaploid wheat is similar to the *turgidum* type branching, thus, the terms genuine branching, *turgidum* type of branching and ramified spike or true spike ramification are used as synonyms. Four-rowed spike that was first recorded in tetraploid *T. turgidum* wheat by Klindworth et al. [17] is a type of HS. In addition to the “true spike ramification”, the term “false-true spike ramification” was recently proposed to describe a particular type of an SS phenotype, that is characterized by elongation of the spikelet rachilla, which bears two florets at basal nodes and SSes at distal nodes [18].

In 1910, E. von Tschermak reported that the spike branching phenotype of *T. turgidum* is under monogenic recessive control, and the gene was designated as *bh*, *branched head* [6]. Results of further research confirmed the recessive mode of inheritance of SS/spike branching in tetraploid *T. turgidum* and *T. durum* wheat species, and revealed the same type of inheritance in diploid *T. monococcum* and hexaploid *T. aestivum* [10, 19–22]. The expression of the trait is modulated by a number of environmental factors [11, 19]. The loci that determined the SS/spike branching phenotype have been localized on wheat chromosomes of homoeologous group two: 2A^m^ in diploid [10], 2A in tetraploid [21, 23], and 2A and 2D in hexaploid wheat [20, 24–26]. The formation of SSes in hexaploid bread wheat is controlled by homoeologous genes *WHEAT FRIZZY PANICLE* (*WFZP*) located on chromosome arms 2AS, 2BS and 2DS, respectively [25]. The structural and functional characterization of the three *WFZP* homoeologous genes has revealed that mutations of *WFZP-D* and *WFZP-A* caused development of supernumerary spikelet phenotypes of MRS, HS, and RS morphotypes in hexaploid bread wheat lines. Poursarebani et al. [27] has suggested that a single amino acid substitution in the AP2/ERF domain of *TtBH-A1*, which represents a mutant allele at the *WFZP-A* locus, causes spike branching in tetraploid *T. turgidum* wheat. In addition to *WFZP-A/TtBH-A1,* other genetic factors are involved in the control of spike branching of the *turgidum* type [28, 29]. Although the *TtBH-A1/wfzp-A* mutant allele at the *WFZP-A* locus is the main genetic determinant of the *turgidum* type of branching/true spike ramification in tetraploid wheat, “false-true spike ramification” phenotype of *T. turgidum* is under the control of a recessive allele at the single *SHR2* locus (*SHAM RAMIFICATION 2*) [30]. The *shr2* gene was placed to chromosome 2AL by genetic mapping [30], but its structure is not defined so far. The “true spike ramification” and “false-true spike ramification” phenotypes have some similarities, development of SSes on an extended rachilla, but it is not clear whether genes *wfzp* and *shr2* are involved in the control of the same developmental processes.

Here, we characterized mutants of diploid wheat *T. monococcum* and tetraploid wheat *T. durum* that show SS/true spike ramification phenotypes; we also identified causative mutations at the *WFZP*-A gene locus, including a novel *wfzp-A.2* mutation, and demonstrated that that the *wfzp-*A/*TtBH-A1* allele may be transferred to *T. durum* via spontaneous hybridization with *T. turgidum*. Moreover, using SEM analysis, we revealed the characteristic features of inflorescence development of the “false-true spike ramification” morphotype and suggested putative functions of the *SHR2* gene. A possible interaction between genes *WFZP* and *SHR2,* which determine the two spike branching phenotypes, was investigated based on evaluation of F_2_ hybrid phenotypes.

## Methods

### Plant material

Diploid wheat material. KT 3–24 is a branched spike induced mutant of *T. monococcum* L. (2*n* = 2*×* = 14, genome formula A^m^A^m^) developed by Dr. K. Yamashita [10]. *T. sinskajae* A. Filat. et Kurk. (2*n =* 2*×* = 14, genome formula A^m^A^m^) accession PI 418587 is characterized by non-branched soft and semi-compact spike. KT 3–24 and PI 418587 were the parents of the F_2_ mapping population for molecular-genetic mapping of the *bh* ^*m*^ gene that determined the mutant spike phenotype [10].

Tetraploid wheat material. *T. durum* Desf. var. *ramosoobscurum* Jakubz. (2*n =* 2*×* = 28, genome formula BBAA) “Vetvistokolosaya” R-107 (R-107 throughout the paper), a semi-dwarf and branched spike accession, is a natural mutant collected in Dagestan, Russian Federation. Branched spike phenotype of R-107 is under the control of a recessive allele at a single gene locus *bh*, located on chromosome 2AS [21].

*T. turgidum* L. К-40750 (2*n =* 2*×* = 28, BBAA) is a branched accession of *T. turgidum* var. *plinianum* originated from Bulgaria and provided by the N.I. Vavilov All-Russian Institute of Plant Genetic Resources (St. Petersburg, Russia).

*T. turgidum L. * PI 67339 (2n = 2× = 28, BBAA) is a tetraploid wheat accession with false-true ramified spike provided by the National Small Grain Collection (NSGC).

*T. durum* Desf. (2*n =* 2*×* = 28, BBAA) Langdon 222 (LD222 throughout the paper) is a standard spiked accession.

We produced three F_2_ populations: (1) R107/K-40750 to assess the allelic relationship of genes that determine *turgidum* type of spike branching (K-40750) and SS/RS phenotype (R-107); this population consisted of 104 plants; (2) PI 67339/R-107 and (3) PI 67339/K-40750 to test possible interaction between the genes that determine branching phenotypes of different types. Populations #2 and #3 consisted of 143 and 162 individuals respectively. F_1_ and F_2_ hybrids were grown under greenhouse conditions of IC&G SB RAS, Novosibirsk, Russia. Spike and spikelet morphology was observed after heading.

### SEM analysis

Developing spikes from lines/accessions with the SS phenotype (KT 3–24, R-107, K-40750, PI 67339) and normal spiked lines, LD222 and PI 418587, were dissected with a scalpel under a binocular microscope (Altami PS0745, “Altami”, St. Petersburg, Russia). Scanning electron microscope, SEM (TM-1000, Hitachi Co. Hitachi, Ltd., Japan), was used to observe the morphological features of the inflorescences as described in Dobrovolskaya et al. [25]. Dissected young spikes were examined under low vacuum conditions (30-50 Pa) and an accelerating voltage of 15 kV.

### Sequencing

Sequences of the *WFZP-A* gene (up to 3000 bp, including the coding part of the genes, promoter regions and 3′-regions) of KT 3–24, R-107, K-407750 (SS/true spike ramification), normal spiked LD222 and PI 418587 lines were obtained by direct Sanger sequencing of PCR products using the BigDye Terminator v3.1 cycle sequencing kit (Applied Biosystems), following the manufacturer’s instructions. Fluorescently terminated extension products were separated using a capillary ABI-3730 Bioanalyzer (Applied Biosystems). All sequences obtained in this study are included in Additional file 1.

All primers used in this study are listed in Table 1.

**Table 1:** Primers used for the *WFZP-A* sequencing and genotyping

Primer Name	Sequence 5′ – 3’	Location
Amplicon sequencing		
WFZP_2A_F1^a^	CATGGGCAAATCGGTTAATG	5′ region
WFZP_2A_R1^a^	TGGATGAGATGGCGAGGTAG	
WFZP_F2^a^	TCTTGTCAGTGGCAGGCATC	5′ region
WFZP_2A_R2^a^	TGGCAGAAGTGAAGTGAGGT	
WFZP_F3^a^	GCTCACAGTCTCAGCAACCA	5’-UTR -CDS
WFZP_2A_R3^a^	CACTGGGCACCGGCATGGAA	
WFZP_2AD_F4^a^	CAGCCAACCTCACTTCACT	CDS
WFZP_2A_R4^a^	GCTAGGGCACCGAAACAAC	
WFZP_F5^a^	ACGACATGGTCGCCTCGT	CDS-3′ region
WFZP_2A_R5^a^	GGATCGGGGTGGATAGATTG	
SSR genotyping		
ssrCS248B13–1F^b^	CTCCAAGAAGATCGAGGTGAACAT	
ssrCS248B13-1R^b^	TTGTTACCCTACCGATGATGTGTG	

^a^[25]

^b^[31]

### Mapping procedure

A previously obtained F_2_ mapping population [10] were genotyped using a *wfzp*-SSR marker, ssrCS248B13–1 [31]. The SSR analysis was performed as described in Dobrovolskaya et al. [25]. The *wfzp-A.2* gene was integrated into 2A^m^ genetic map reported by Amagai et al. [10].

## Results

**Fig. 1:**
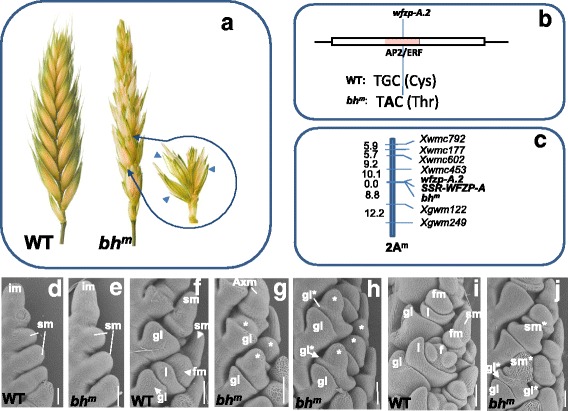
Spike branching in diploid *T. monococcum* wheat. **a**, Illustration of the spike structure in the *T. monococcum* KT 3–24 (middle), harboring short branch-like structures (right) with supernumerary spikelets (indicated with blue arrows) and *T. sinskajae* PI 418587 (a WT-spike) (left). **b**, Schematic representation of the *WFZP* gene structure and the mutation identified. The light red box indicates the AP2/ERF domain. **c**, A partial 2A^m^ molecular-generic map showing collocation of *bh* ^*m*^, *wfzp*-*A.2* and SSR*-WFZP-A* (ssrCS248B13–1 from Dobrovolskaya et al. [31]) on 2A^m^. Genetic distances are given in cM. **d**-**j**, Scanning electron microscopy images of KT 3–24 (*bh* ^*m*^) and PI 418587 (WT) inflorescences at various developmental stages. **d** and **e**, Spikelet differentiation stage in the wild type (**d**) and *bh* ^*m*^ (**e**). F, Early floret differentiation stages when the spikelet meristem produces the FM in the wild type. **g** and **h**, Differentiation of secondary AxMs (indicated by asterisks) in the *bh* ^*m*^ mutant. **h** and **j**. The development of glumes (gl*) and FM*s by secondary AxMs in the *bh* ^*m*^ mutant. **i**, Floret differentiation stage showing differentiated floral organs in the WT-inflorescence. **j**, The development of ectopic spikelets in *bh* ^*m*^. Bars = 100 μm. **f**, floret with floret organ primordia; fm, floret meristem; gl, glume; im, inflorescence meristem; l, lemma, sm, spikelet meristem; sm*, spikelet meristem of an ectopic spikelet

**Fig. 2:**
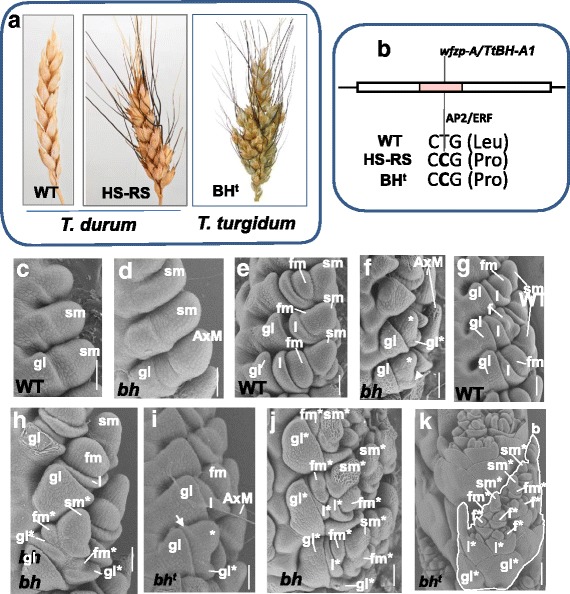
Ramified spike and *turgidum*-type of spike branching in tetraploid wheats. **a**, Illustration of structure of standard spike (WT) in *T. durum* LD222 (left), ramified spike (indicated as gradation from horizontal spikelets, HS, to ramified spike, RS) in *T. durum* R-107 (middle) and the *turgidum*-type of spike branching (indicated as branched head, BH^t^) in *T. turgidum* K-40750 (right). **b**, Schematic representation of the *WFZP-A* gene structures and the mutation identified. The light red box indicates the AP2/ERF domain. **c**-**k**, Scanning electron microscopy images of LD222 (WT), R-107 (*bh*) and K-40750 (*bh* ^*t*^) inflorescences at various developmental stages. **c** and **d**, Spikelet differentiation stage in the wild type (**c**) and *bh* (**d**). **e**, Early floret differentiation stages when the spikelet meristem produces FMs in WT. **f**, Differentiation of secondary AxMs (indicated by asterisks) in the *bh* mutant. **h** and **i**, The development of glumes (gl*, indicated by arrows) and floret meristems (fm*s) by secondary AxMs in the *bh* and *bh* ^*t*^ mutants. **g**, Floret differentiation stage in the WT-inflorescence. **h**, development of an ectopic spikelet at place of a basal floret. **j** and **k**, Development of ectopic branch-like structures (**b**) in the *bh* and *bh* ^*t*^ mutants. Bars = 100 μm. **f**, floret with floret organ primordia; fm, floret meristem; gl, glume; im, inflorescence meristem; l, lemma, sm, spikelet meristem; sm*, spikelet meristem of an ectopic spikelet

**Fig. 3:**
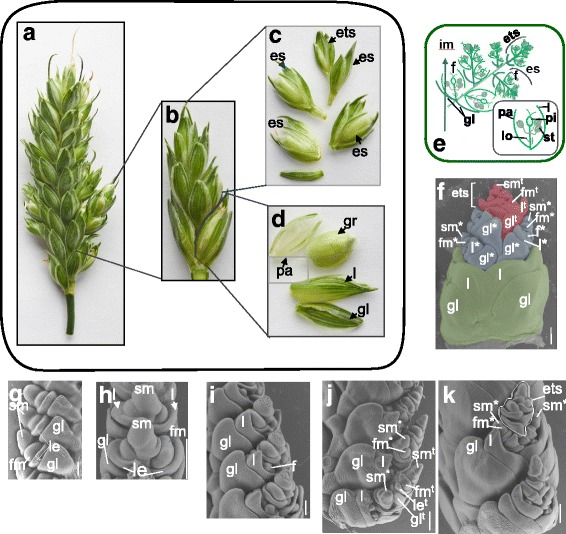
False-true spike ramification in tetraploid *T. turgidum* wheat. **a**-**d**, Illustration of the spike structure in the *T. turgidum* PI 67339 (**a**), harboring elongated spikelets (**b**) with grains in the basal part (**d**) and ectopic spikelets in the distal part (**c**). **e**, Schematic illustration of an altered spikelet structure of the false-true spike ramification phenotype, showing locations of florets (f ), ectopic spikelets (es) and an ectopic terminal spikelet (ets), and structure of a WT-floret (right, bottom). **f**, Scanning electron microscopy image of an elongated spikelet dissected from the false-true ramified spike, showing the spikelet glumes (gl) and lemmas (l) of a basal pair of florets (in green), three developing ectopic spikelets at the floret differentiation stage (in gray) and an ectopic terminal spikelet (ets) at the early floret differentiation stage (in red). **g**-**k**, SEM images of the PI 67339 inflorescence at various developmental stages. **g** and **h**, Early floret differentiation stage. **i**, beginning of the floret differentiation stage, when the basal floret begin to differentiate. **j** and **k**, Development of ectopic spikelets in the distal part of altered spikelet. Bars =200 μm. f, floret with floret organ primordia; f*, f^t^, floret of an ectopic spikelet (es) and an ectopic terminal spikelet (ets), respectively; fm, floret meristem; fm*, fm^t^, floret meristem of es and ets, respectively; gl, glume; gl*, gl^t^, glume of es and ets, respectively; l, lemma; l*, l^t^, lemmas of es and ets, respectively; sm, spikelet meristem; sm*, sm^t^, spikelet meristems of es and ets, respectively

Standard wheat inflorescence, the spike, consists of sessile spikelets that are directly attached to the spike rachis in the distichous order, one spikelet per a rachis node. The spikelet is a short branch, bearing florets; a spikelet consists of two glumes and 2–5 florets arranged along a spikelet rachilla. The main axis in wheat is determinant and terminates with a terminal spikelet, although in diploid *T. monococcum,* the terminal spikelet is rudimentary or missing [32–34]. Here, we characterized lines and accessions of diploid and tetraploid wheats with non-standard spike morphology that are characterized by the development of SSes and/or extension and branching of a spikelet rachilla. The SS group is heterogeneous and comprises lines with different morphotypes: horizontal spikelets, genuine branching, and false-true spike ramification (Figs. 1, 2, 3).

### *HS (horizontal spikelets)* and *true spike ramification/genuine branching phenotypes*

Diploid *T. monococcum* L. wheat. KT 3–24, an induced fertile mutant of *T. monococcum*, is characterized by a genetically stable SS trait. Additional spikelets developed on short branches (Fig. 1a). At the earliest stage of development, KT 3–24 inflorescence developed normally (Fig. 1e); inflorescence meristem produced primary axillary meristems (AxMs) that first initiated glume primordia and then gave rise to secondary AxMs (Fig. 1g). In the wild type (WT), secondary AxM (floral meristem in a WT inflorescence) first produced lemma and palea, and then formed floral organs (Fig. 1d, f), but in KT 3–24 inflorescence, secondary AxMs formed glume primordia (Fig. 1f) and then generated the other spikelet organs (Fig. 1j), and thus ectopic spikelets developed at the location of florets of a primary spikelet. Several secondary AxMs of a primary spikelet developed into ectopic spikelets and, as a result, a branch-like structure formed. Glumes of primary spikelets remained rudimentary and could be distinguished only at early stages of spike development (Fig. 1h, j). Ectopic spikelets developed as WT spikelets and produced florets. The meristems of ectopic spikelets formed at 90° to normal spikelets.

The feature of the KT-3-24 inflorescence development were similar to those of previously described bread wheat SS mutants [25] and *bh1* mutants of *T. turgidum* [27], namely, 1) development of an ectopic spikelet at the location of the floret of the primary spikelet; 2) development of ectopic spikelet meristems at 90° to normal spikelets; 3) in an ectopic branch-like structure, the basal florets of the primary spikelet were always replaced by ectopic spikelets; 4) rudimentary glumes of the primary spikelets were visible only at the early stages of inflorescence development.

Suggesting these features of inflorescence development together with the location of gene *bh* ^*m*^ responsible for the mutant phenotype on the 2AS molecular-genetic map [10], we hypothesized that *bh* ^*m*^ is a mutant allele at the *WFZP* gene locus in diploid *T. monococcum* wheat. A set of *WFZP*-A sequence-specific primers [25] were used for sequencing of *WFZP* in *T. monococcum* (Table 1). In line KT 3–24, a non-synonymous substitution, T**G**C (Cys) to T**A**C (Thr), was found in the AP2/ERF functional domain of *WFZP* (Fig. 1b). The *WFZP* sequences of F_2_ individuals from the KT 3–24/PI 418587 cross were determined. It was found that all the plants with the mutant phenotype had the mutant allele. Moreover, the CS248B13 microsatellite marker (Table 1) developed on the basis of sequence analysis of the *WFZP-A* locus [31] was applied to genotype the F_2_ plants. *WFZP* and CS248B13 were mapped on chromosome 2AS map and they did not recombine with *bh* ^*m*^ based on 94 F_2_ individuals (Fig. 1c). Thus, the mutant phenotype of line KT 3–24 resulted from the *WFZP* mutation. This novel allele was designated as *wfzp*-A.2, in accordance with *wfzp*-A.1, a frameshift mutation in bread wheat [25], and *wfzp-A/TtBH-A1*, a non-synonymous substitution in the AP2/ERF functional domain in *T. turgidum* tetraploid wheat [27].

Tetraploid *T. durum* wheat. R-107, a natural mutant of *T. durum*, is characterized by the development of a sessile SSes at spikelet nodes (under greenhouse conditions) and on extended rachilla (more frequently under field conditions). Its phenotype was similar to the horizontal spikelet phenotype of bread wheat, occurring when two or three spikelets are in a horizontal position at a spike rachis node, and to the four-rowed spike (FRS) phenotype of tetraploid *T. turgidum* wheat but rather different from the typical *turgidum* spike, which has more SSes attached to a long extended rachilla (Fig. 2a). SEM analysis of inflorescence development reveal that the first deviation from the standard scheme of development of R-107 occurred at the stage when the floret meristems produced lemma primordia (Fig. 2e) in a WT spikelet, the first floral organs, in the WT-inflorescence. By contrast, in line R-107, two glume primordia become apparent (Fig. 2f), and, then secondary AxMs produced the other spikelet organs (Fig. 2h, j). Therefore, ectopic spikelets developed exactly at the place of florets, as revealed in SS mutants of diploid (K-3-24) and hexaploid wheat [25]. Within a spikelet, only a basal floret(s) could be replaced by a spikelet (Fig. 2h), resulting in development of the HS supernumerary spikelet phenotype (FRS phenotype in tetraploid wheat); often, ectopic spikelets that developed distally (3rd and more distally located) did not fully develop, this arrangement also resulted in HS and FRS. We compared features of the R-107 inflorescence development with those of *T. turgidum* L. K-40750, which is characterized by the typical *turgidum* type of branching (Fig. 2a). In general, they were similar e.g., development of an ectopic spikelet at the location of florets at 90^0^ to WT spikelets (Fig. 2j-k) and conformed to the characteristic features of line KT-3-24 listed above (Fig. 1g, h, j).

In hexaploid, diploid (this study), and tetraploid *T. turgidum* wheats, the SS/spike branching phenotypes resulted from *wfzp-*mutations, and *WFZP*-A was chosen as a candidate gene for the SS phenotype of line R-107. The *WFZP*-A gene was sequenced in line R-107 and standard spiked LD222. We found a non-synonymous substitution, C**T**G (Leu) to C**C**G (Pro), in the AP2/ERF domain of the *WFZP-A* gene of line R-107. This allele is not novel and has been found in branched accessions of *T. turgidum* [27], near-isogenic lines that have the four-rowed spikelet FRS; and RS phenotypes of *T. turgidum* [28, 29]. The *WFZP*-A gene was also sequenced in a branched accession *T. turgidum*, K-40750. As expected, K-40750 possessed the *wfzp-A*/*TtBH-A1* mutant allele. This finding was supported by results of the complementation test: the R-107 line was crossed with K-40750, and phenotypes of the hybrids were examined. All F_1_ plants had the branched spike phenotype intermediate between the parental types; the F_2_ plants segregated into the FRS and RS/*turgidum* types of branching, and both F_1_ and F_2_ showed the SS-phenotype.

Thus, the *wfzp*-mutations in diploid, tetraploid and hexaploid wheat species lead to similar abnormal development of the inflorescence and resulted in SS-phenotypes: MRS, HS (FRS), and RS/genuine branching*/turgidum* type of branching.

### False-true ramification of a spike

*T. turgidum* PI 67339 has an SS-phenotype different from those determined by the *wfzp*- mutations. First, the trait was described as sham ramification (SHR), which is characterized by formation of an extended spikelet axis (rachilla) with attached florets [30]. The spike phenotype of line PI 67339 is controlled by a single recessive gene designated as *shr2* (*sham ramification* 2) by Amagai et al. [30]. Further careful observation revealed that the SHR phenotype of PI 67339 is different from “sham-ramification” of *T. jakubzineri*, with florets at basal and apical nodes of an elongated spikelet rachilla [18]. The elongated rachilla of *T. turgidum* PI 67339 bears florets at basal nodes while containing spikelets at the apical nodes. Amagai et al. [18] proposed a new status of PI 67339 ramification, “false-true ramification”.

Under greenhouse conditions (Novosibirsk, 2014—2017), the PI 67339 line was characterized by incomplete penetrance (~ 90%). In contrast, all *wfzp*-mutants of di-, tetra- and hexaploid wheat species showed 100% penetrance. We found that elongated spikelets of line PI 67339 formed at rachis nodes of the middle part of the spike (Fig. 3a). The glumes of elongated spikelets were fully developed and looked like those of WT spikelets. By contrast, glumes of branch like structures of the *wfzp*-mutants were always rudimentary ([25], results of present study). The elongated spikelet of PI 67339 was subtended by two glumes and consisted of two fertile florets that formed grains in the basal part of the spikelet, and several SSes attached to the extended spikelet rachilla (Fig. 3c-e). In these SSes, either one or two florets were fertile.

At the early stages of PI 67339 inflorescence development, the apical meristem produced primary AxMs (spikelet meristems) that first formed spikelet organs, glumes, and then formed secondary AxMs (Fig. 3g-h). Next, lemmas, that were first produced by secondary AxMs became visible, after which the other organs of two basal florets developed (Fig. 3i). Although the two basal florets of the elongated spikelet developed in agreement with the standard scheme of development (Fig. 2c, e, g, showing inflorescence development of line LD222: a standard spiked tetraploid wheat cultivar), further spikelet development was disrupted (altered). Secondary AxMs (correspond to the 3rd—5th floret meristems in the WT spikelet) produced glume primordia and spikelet organs at the place of florets until primary AxMs converted to the terminal spikelet meristem and formed a terminal spikelet oriented 90° to the ectopic spikelets (Fig. 3f, j, k). Normally,  the wheat spikelet meristem is indeterminate and continually produces floret meristems. In line PI 67339, primary axillary (spikelet) meristem first produced two florets (this situation conforms to the standard scheme of development), then produced 2—3 spikelets at the places of florets (in the way similar to that of - *wfzp-*mutants) and finally formed a terminal spikelet at 90° to previously developed ectopic spikelets.

Thus, inflorescence development of line PI 67339 has some features in common with that of *wfzp*-mutants, namely, development of ectopic spikelets at the place of the floret at 90° to normal spikelets. The PI 67339 false-true ramification phenotype is under the control of the recessive *shr2* gene, which was mapped to 2AL [31]. We crossed line PI 67339 (*shr2*) with R-107 (HS/RS phenotype under the *wfzp-A/TtBH-A1* control) and K-40750 (the *turgidum* type of spike branching under the *wfzp-A/TtBH-A1* control) and analyzed phenotypes of F_1_ and F_2_ hybrids to examine a possible gene interaction.

### The PI 67339 × R-107 cross

**Fig. 4:**
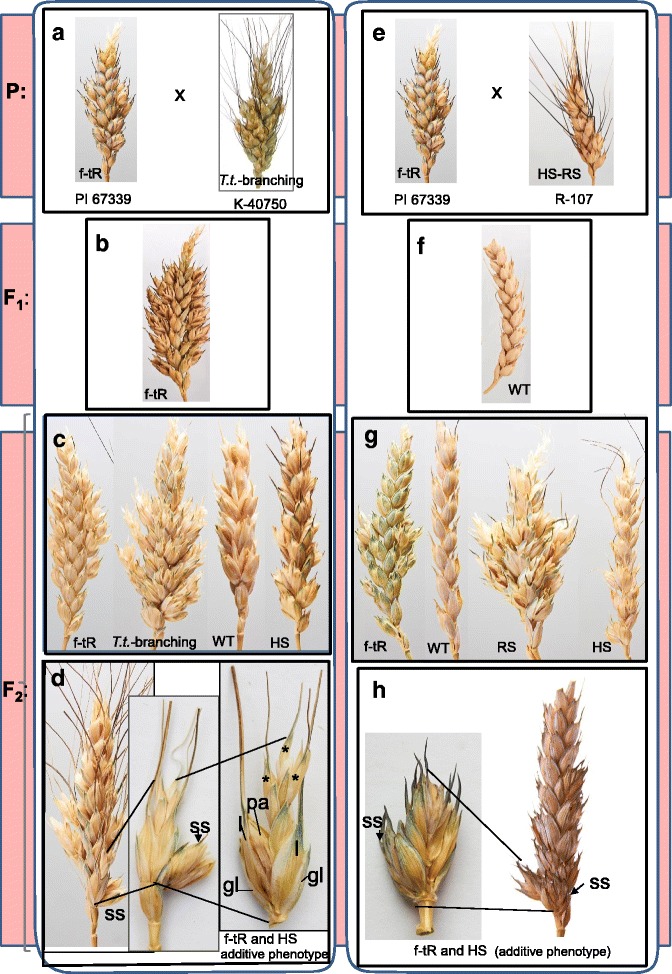
Spike phenotypes of F_1_ and F_2_ hybrids derived from the crosses of *T. turgidum* PI 67339 (*shr2*), *T. durum* R-107 (*wfzp-A/TtBH-A1*) and *T. turgidum* K-40750 (*wfzp-A/TtBH-A1*). **a** to **d**, spikes of the parental lines (**a**), F_1_ (**b**) and F_2_ (**c**, **d**) hybrids derived from the PI 67339/K-40750 cross. **e** to **h**, spikes of the parental lines (**e**), F_1_ (**f**) and F_2_ hybrids (**g**, **h**) derived from the PI 67339/R-107 cross. **c** and **g**, F_2_-spikes, representing different phenotypic classes: false-true ramification (f-tR), *turgidum* type of branching (*T.t.*- branching), ramified spike (RS), horizontal spikelet (HS), standard or WT-spike (WT). D, F_2_-spike of additive phenotype (left) harboring an extra spikelet (supernumerary spikelet, SS) (middle) and an elongated spikelet of f-tR-type (right) at one rachis node. H, A spike of additive phenotype (right) with the SS and f-tR-type spikelets at one rachis node (left). gl – glumes, l – lemma, pa – palea, asterisks indicate ectopic spikelets

F_1_ plants from the PI 67339/R-107 cross showed the WT spike phenotype (Fig. 4f). F_2_ hybrids could be subdivided into several phenotypic classes: (#1) the standard spike; (#2) the HS/FRS SS-phenotype; (#3) the true spike ramification (ramified spike, RS), when SSes developed at an extended rachilla (both #2 and #3 had the R-107 parental phenotype); (#4) false-true spike ramification, (f-tR, the PI 67339 parental phenotype); (#5) an additive phenotype, a combination of both parental phenotypes, then sessile SSes and an extended spikelet of the f-tRS type emerged at one rachis node (Fig. 4h; Table 2). The segregation ratio of **40** R-107 parental phenotype (BH) F_2_ plants, including classes #2, #3, and #5, to **103** non-BH F_2_ plants (classes #1 and #4) conformed to the 1:3 segregation (χ^2^ = 0.67), which confirmed monogenic genetic inheritance of *bh*. The segregation ratio of 22 f-tR (classes #4 and #5) to 121 non-f-tRS deviated from 1:3 (χ^2^ = 7). The ratio of **4** additive phenotype plants (#5) to **139** plants of the other phenotypic classes (# 1–4) fitted to 1:15 segregation (χ^2^ = 2.7, *P* = 0.05–0.025). These results indicated that genes *bh* (*wfzp-A*) and *shr2* are inherited independently and may belong to different pathways; otherwise, we would have observed dominance of one of the parental phenotypes or appearance of a new (enhanced) phenotype.

**Table 2:** Segregation data in F_2_ populations

Cross	F_2_						
	NS (#1)	HS (FRS) (#2)	RS (#3)	f-tR (#4)	HS and f-tR (#5)	SHR (#6)	Total
PI67339/K40750	26	7	12	103	4	10	162
PI67339/R-107	85	32	4	18	4	–	143

*HS* horizontal spikelet, *FRS* four-rowed spike, *RS* ramified spike, *f-tR* false-true ramification, *NS* normal spike, HS and f-tR additive phenotype, *SHR* sham ramification (plants showing extended spikelet rachilla harboring florets)

### *The PI 67339* × *K*-40750 *cross*

Although the *wfzp-A/TtBH-A1* mutant allele is a major genetic factor that determined the SS phenotypes of lines R-107 and K-40750, we obtained different results from the PI 67339/R-107 and PI 67339/K-40750 crosses*.* The F_1_ hybrids from the PI 67339/ K-40750 cross had the PI 67339 parental spike type, f-tR, with more severe trait manifestation (Fig. 4b). F_2_ hybrids segregated into several phenotypic classes (Fig. 4c-d, Table 2). In addition to the above-mentioned classes ##1—5, a new phenotypic class (#6), showing spikes with extended spikelet rachilla containing numerous florets (sham ramification) was detected (Additional file 2). The segregation ratio 107 f-tR to 55 non— f-tR deviated from 3:1 (χ^2^ = 6.9) and indicated the dominant mode of inheritance in this cross. Segregation of **23** BH F_2_ plants (classes #2, #3, and #5) into **139** non-BH (χ^2^ = 10.8) also deviated from 1 to 3 monogenic inheritance, and additive phenotype (Fig. 4d) was observed in 4 of 162 F_2_ plants, which deviated from the 1:15 segregation ratio (χ^2^ = 3.9).

Zhang et al. [29] demonstrated that in addition to *wfzp-A/TtBH-A1*, another genetic factor(s) is involved in the genetic control of the *turgidum* type of spike branching. These factors determine elongation of a spikelet rachilla and development of a RS (ramified spike). Phenotypes of lines R-107 and K*-*40750 differed in the length of the rachilla*,* K*-*40750 has long rachillas with numerous SSes, whereas R-107 possesses either HS/FRS or a short-rachilla (SHR) phenotype. Our results suggested that the *shr2* gene may interact with the genes that participate in the development of the RS phenotype (*turgidum* branching), and these gene(s) may share similar functions with the *shr2* gene. The presence of the additive phenotypic class (HS and f-tR) among the F_2_s from the PI 67339/K40750 cross confirmed the results of the PI 67339/R-107 cross, indicating independent inheritance of *shr2* and *wfzp-A/TtBH-A1* and that the genes may belong to different genetic pathways.

## Discussion

Wheat lines with SSes is very heterogeneous group, which comprises various non-standard morphotypes: (1) multirow spike (MRS) with numerous SSes at a rachis node; (2) horizontal spikelet (HS), including a triple spikelet, and a similar phenotype in tetraploid wheat species called four rowed spikelet (FRS); (3) genuine branching (GB) or ramified spike (RS) or *turgidum* type of spike branching (synonyms); (4) vertical spikelets (VS) or pared spikelets, which are characterized by the formation of the second spikelet immediately adjacent to and directly below a typical single spikelet; (5) false-true spike ramification (f-t SR) with florets in the basal part and spikelets in the apical part of the elongated spikelet rachilla. Dobrovolskaya et al. [25] have demonstrated that mutations in *WFZP-A,* and *-D* genes result in HS (including the triple spikelet), MRS and RS phenotypes in hexaploid wheat. Poursarebani et al. [27] have shown that the *TtBH-A1* mutation (a mutation at the *WFZP*-*A* gene locus) is responsible for development of the *turgidum*-type of spike branching in tetraploid *T. turgidum* wheat. Zhang et al. [28, 29] have developed near isogenic lines with FRS and long ramified spike phenotypes based on a cross of branched *T. turgidum* and standard spiked lines, and demonstrated that, in addition to *TtBH-A1*, other genetic factors are involved in the genetic control of the SS phenotype with the extended rachilla, including the RS-phenotype. Among these factors, Zhang highlighted a dominant gene that is also localized to chromosome 2A [28, 29]. Although *wfzp*-mutations are responsible for MRS, HS, triple-spikelet, and RS (GB) SS phenotypes, the VS or pared-spikelet phenotype is under the control of another gene. Boden et al. [34] have revealed that *Photoperiod-1* (*Ppd-1*), a pseudo-response regulator gene that controls photoperiod-dependent floral induction, has a major inhibitory effect on paired spikelet formation by regulating the expression of *FLOWERING LOCUS T* (*FT*).

In the present study, we examined an induced SS-mutant with the branched spike (development of SSes on short branches) in diploid wheat *T. monococcum* and found that the mutant phenotype resulted from a missense mutation in the AP2/ERF functional domain of *WFZP-A*. This novel allele was designated as *wfzp-A.2.* Spontaneous mutants with spike branching have not been found in diploid wheat species [9].

At the tetraploid level, *T. turgidum* forms with spike-branching have been well known for a long time [4]. Poursarebani et al. [28] demonstrated that the phenotype of 30 branched *T. turgidum* accessions is associated with the same mutant allele *TtBH-A1* at the *WFZP-A* gene locus; this finding is suggestive of this allele’s monophyletic origin. Distribution areas and ecological characteristics of unbranched *T. turgidum* convar*. turgidum* and branched *T. turgidum* convar. *compositum* (L.f.) Filat. accessions in general coincide; with an exception of the Tibetan ecotype, all accessions of this ecotype are unbranched [4]. Consequently, the *TtBH-A1* mutation may have arisen early in the *T. turgidum* evolutionary history and then has been spread across modern habitats of this species.

On the other hand, in *T. durum* tetraploid wheat, branched spiked forms are rare [4]. In 1952, M.M. Jakubziner found branched forms in two cultivars of *T. durum*: Akmolinka 5 and Hordeiforme; these forms were designated as *ramosohordeforme* and *ramosoapulicum* [8]*.* Two original forms of durum wheat with a branched spike, var. *ramosohordeforme* and var. *ramosoapulicum,* were found by V.F. Dorofeev during an expedition to Transcaucasia (1961—1964) under the natural conditions of wheat growing in local mixed populations, where bread wheat, *T. durum* and *T. turgidum* were grown side by side [8]. Dorofeev suggested that the branched forms of *T. durum* might have originated from spontaneous hybridization between branched *T. turgidum* accessions and standard spiked *T. durum*, which were grown side by side in Transcaucasia. We characterized the spontaneous SS mutant of *T. durum*, R-107, which was found in Dagestan, Russia, and demonstrated that its mutant phenotype is determined by the *wfzp-A/TtBH-A1* mutant allele*,* which is responsible for the branching spike phenotype of *T. turgidum* convar. *compositum* (L.f.) Filat. This finding confirmed Dorofeev’s assumption that the branched phenotype *of T. durum* grown in Transcaucasia – or in any regions where *T. turgidum* and other wheat species crossable with *T. turgidum* grow side by side may have originated from *T. turgidum* via spontaneous hybridization. It should be noted that the R-107 line showed a phenotype that is more similar to FRS than to the typical *turgidum* spike branching type. This finding means that the genetic background is important for expression of the *turgidum* type of spike branching.

Now that the *wfzp* mutants were characterized in detail in di-, tetra-, and hexaploid wheats, we can make some conclusions regarding common characteristics of their phenotypes and developmental features that can help to predict whether any new uncharacterized SS-mutant phenotype may be a result of a *wfzp*-mutation. Even though *wfzp* mutations caused several SS morphotypes mentioned above, all these morphotypes have common characteristics: location of ectopic spikelets at the place of florets at 90° to a WT-spikelet or an ectopic spikelet of the previous order as well as formation of rudimentary glumes that are visible only at the early stages of development. Under the influence of weak *wfzp* mutations, only one SS develops at a rachis node of the lower part of a spike [25, 35], and these spikelets are always positioned at an angle to a WT-spikelet. It is also noteworthy that both weak and severe *wfzp* mutations cause replacement of basal florets by ectopic spikelets, whereas more distally located florets within the altered spikelet may develop normally in weak *wfzp* mutants [25, 36]. Independently of the ploidy level, all the characterized *wfzp*-mutants of wheat were fertile.

The phenotype of *wfzp* mutants is similar to phenotypes of barley *com2* [27], Brachypodium *moc1* [36], rice *fzp* [37] and maize *bd1* [38] mutants. The expression pattern and function predicted by means of *fzp*-mutant phenotypes were similar*.* Bai et al. [39] demonstrated that in rice *FZP* plays an important role in the regulation of ABCDE floral organ identity genes. *FZP* controls panicle branching and spikelet formation by regulating *RFL*/*ABERRANT PANICLE ORGANIZATION 2* (*APO2*). Overexpression of *FZP* severely represses AxM formation and outgrowth of secondary branches in the panicle, and it positively regulates the expression of a subset of the class B and E genes, suggesting that *FZP* may specify floral organ identity by regulating the related OsMADS-box genes [39].

At present, *wfzp*-mutants and VS lines are the most studied among SS-wheats [25, 27, 34], whereas sham ramification and false-true spike ramification traits are not well studied so far. False-true ramification line PI 67339 was examined here by SEM analysis and a classical genetic approach. We found that inflorescence development of the PI 67339 line and that of SS lines, determined by *wfzp* mutations, have some common features, namely, development of ectopic spikelets (at the place of florets), which are oriented at 90° to WT-spikelets. Nonetheless, the earliest events during spikelet development of these two morphotypes were different: in PI 67339, development of the 1^st^ and 2^nd^ florets conforms the standard scheme of wheat inflorescence development, but in the *wfzp*-mutants, abnormal development was observed earlier, and ectopic spikelets developed always at the place of floret(s) in the basal part of a spikelet. The *FZP* gene and its orthologs in cereals participate in the establishment of floral meristem identity, and *fzp* mutations affect early events during spikelet development [25, 27, 36–38]. The *shr2* mutant phenotype suggests that *SHR2* is required for establishing floret meristems at a later stage of spikelet development, during the development of the 3^rd^ to 5^th^ florets. Results of tests for gene interaction, namely, the presence of an additive phenotype in F_2_ hybrid populations produced by crosses of R-107 (*wfzp-A*/*bh1*), K-40750 (*wfzp-A*/*bh1*), and PI 67339 *(shr2*), suggest that the *WFZP* and *SHR2* genes function independently in different processes during spikelet development.

Wheat has indeterminate spikelet meristems, SMs, and the wheat spikelet carries multiple florets. The number of fertile florets per spikelet was determined by the number of floret primordia and of florets with hypoplasia. Indeterminacy of the SM is not affected by polyploidization and is a characteristic feature of the spikelet of di-, tetra- and hexaploid wheats [40]. In florets of the distal portion of the spikelet, two types of hypoplasia have been observed: (1) floret organs are differentiated, but they are sterile and aborted (e.g., tetra- and hexaploid wheats), or (2) floret meristem initiates but no floral organs develop (in diploid wheat) [40]. In *wfzp*-mutants, the primary AxM (corresponds to SM in a WT spikelet) is indeterminate and produces numerous secondary AxMs (correspond to FMs in a WT-spikelet) that either form ectopic spikelets or, due to hypoplasia in the distal part of a branch-like structure, remain underdeveloped. Indeterminacy is a characteristic feature of branch-like structures of *fzp* mutants in wheat (*wfzp*), barley (*com2*), rice (*fzp*), and maize (*bd1*). Nonetheless, in the false-true spike ramification line PI 67339, the altered extended spikelet is determinate and possesses an ectopic terminal spikelet that developed at 90° to the other ectopic spikelets; this arrangement suggests that mutations in the *SHR2* gene may alter the SM fate. Although the *shr2* gene has not been cloned yet, we can suggest its function(s) on the basis of its mutant phenotype and the features of inflorescence development. The gene may be involved in the maintenance of floral meristem identity during spikelet development, when the 3^nd^ – 4^th^ (5^th^) florets differentiate; moreover, *shr2* may determine the spikelet meristem fate. Our results suggest that *WFZP* and *SHR2* are independently inherited and probably “sit” on different regulatory pathways, whereas still unidentified gene(s) that are involved in the control of the *turgidum* spike branching phenotype in addition to the major *wfzp-A/TtBH-A1* gene may interact with *shr2* and share some functions in the inflorescence development*.*

## Conclusions

Accordingly, SS mutants represent an important genetic tool for research on the development of the wheat spikelet and for identification of genes that control meristem activities. Further studies on different non-standard SS morphotypes and wheat lines with altered spike morphology will allow researchers to identify new genes that control meristem identity and determinacy, to elucidate the interaction between the genes and to understand how these genes, acting in concert, regulate the development of the wheat spike. SS wheats lines have been attracting attention of breeders for many years; but their potential has not been realized yet.

## Additional files

Additional file 1: Sequences of *WFZP-A* of diploid and tetraploid wheat species. (PDF 43 kb)

Additional file 2: Spike with extended spikelet rachilla containing numerous florets. (PDF 170 kb)
