# Supplementary material for: Genes WHEAT FRIZZY PANICLE and SHAM RAMIFICATION 2 independently regulate differentiation of floral meristems in wheat
Source: BMC Plant Biol. 2017 Dec 28;17(Suppl 2):252. doi: 10.1186/s12870-017-1191-3 (PMC5751757; doi:10.1186/s12870-017-1191-3)
Supplement: Supplementary file 1 — Sequences of WFZP-A of diploid and tetraploid wheat species. (PDF 43 kb) [file 12870_2017_1191_MOESM1_ESM.pdf]

Sequences of *WFZP* in diploid and tetraploid wheat species.

CDSs are in green.

>*T. monococcum* KT3-24

acacatacacacacacatatatagctatatgtttgactggcacaacattctcctcatctctttggtcctaagattag
ggcacacaaatccaaacacaaggtgcatacatacaaacttgcagtgagctttccattccattccctccttttactcc
tagaattaaacaagtgcagcgcatgcatgtccctcccctcctcccctacctcgccatctcatccacagtgctctcac
tccttctagcttctccctcctccgagctccccatttaaataccaccctgcctccctccatagtgagcactacacact
agaagctcacagtctcagcaaccacctcctcaacctaagctagctagctcacacctcagagctcaagctaggcggga
gcagtagtatagtagccagccaacctcacttcacttctgccATGAGCTCTCGCAGCAGCAGCGGCGGCGGCGGTGCC
TCCCAGATGATGGCCTTCTCGGAGCATTCGCTGCCGAAGCCGATCGCCGGTCACCCGCAGCCGCAGCCGTCCCCGCC
GTCGTCGCCGAGCGAGCGGCCGGCGGCGCGCGGCAGGCGGCGCGCGCAGGAGCCCGGGCGCTTCCTGGGCGTGCGCC
GGCGGCCGTGGGGCCGGTGCGCGGCCGAGATACGCGACCCGACCACCAAGGAGCGGCACTGGCTCGGCACATTCGAC
ACGGCGCAGGAGGCCGCCCTGGCCTACGACCGCGCCGCGCTCTCCATGAAGGGCGCGCAGGCGCGCACCAACTTCGT
CTACGCGCACGCCGCCTACAACAACTACCCGCCCTTCCTCGCGCCGTTCCACGCGCAGCACCAGCCCGCCGCCTACG
CCGCGTCCTCGGCCATGCCGTACGGCGGCCAGCAGCAGCACGCGGGCGCGGGGCCGCCGCACATTGGCAGCTCGTAC
CACCACGGCCACGGCTACCACCAGCAGGGCCCGGGCGAGTGCTCCATGCCGGTGCCCAGTGCCGCGGATCACGGCGC
CAGCGGCCCGATGGACGTGCGCGGCAGCAGCGGCCACGACTTCCTCTTCCCCAGCGCCGACGACAACTCCGGGTACC
TGAGCAGCGTGGTGCCGGAGAGCTGCCTCCGGCCCCGCGGCGGCGACCTGCAGGACGCGCGGCGCTACTCCGTGTCC
GACGCCGACGCCTACGGGCTGGGCCTCCGGGAGGACGTGGACGACCTGGCGACGATGGTGGCCGGCTTCTGGGGCGG
CGCCGACGCGCCGTACGGCGGCGGCCACGACATGGTCGCCTCGTCGCAGGGCTCGGACAACGGCTACTCCCCCTTCA
GCTTCCTCTCCCACTGAaacggacgcgctggcattgctcttagcctcttacgtactcagtggtctgctgtgcccacc
gtcggccattagctaggtactgtttgcttcgtgagttcgatcgatcgatcatagtagaagatcgtgaagaagagtgg
ctttgttgtttcggtgccctagcttagcggatgctcacatccatgtacttaggttctttcaattagcagcactatta
ctacgtactactagtactagtaagttttcatgcatgcacgagtgacatgcttaagttatctctctctaaaattatta
cagcctctctctaattagttagtcgtgtggaattaaagtggcggatcgagagtactcgagaggcccgggaatgtagt
acgcacacgcacactgttgttgatccttgaaagcttcagttttgggttgtagttttgtttgattcagataggccggc
cggaggaacatgtcaaagtacgtactctcctacgtacttacctcaaggaagaagataaggggaatagtacacggacc
acgtactagacgtagtactacccattgacagttcaacggaaaggtagatgttcaggacaagtgtcaagcaggcgacc
tcctttttttttcttgggtcgccaggtacttggtgctga

>*T. monococcum* KT3-1

tatatagctatatgtttgactggcacaacattctcctcatctctttggtcctaagattagggcacacaaatccaaac
acaaggtgcatacatacaaacttgcagtgagctttccattccattccctccttttactcctagaattaaacaagtgc
agcgcatgcatgtccctcccctcctcccctacctcgccatctcatccacagtgctctcactccttctagcttctccc
tcctccgagctccccatttaaataccaccctgcctccctccatagtgagcactacacactagaagctcacagtctca
gcaaccacctcctcaacctaagctagctagctcacacctcagagctcaagctaggcgggagcagtagtatagtagcc
agccaacctcacttcacttctgccATGAGCTCTCGCAGCAGCAGCGGCGGCGGCGGTGCCTCCCAGATGATGGCCTT
CTCGGAGCATTCGCTGCCGAAGCCGATCGCCGGTCACCCGCAGCCGCAGCCGTCCCCGCCGTCGTCGCCGAGCGAGC
GGCCGGCGGCGCGCGGCAGGCGGCGCGCGCAGGAGCCCGGGCGCTTCCTGGGCGTGCGCCGGCGGCCGTGGGGCCGG
TACGCGGCCGAGATACGCGACCCGACCACCAAGGAGCGGCACTGGCTCGGCACATTCGACACGGCGCAGGAGGCCGC
CCTGGCCTACGACCGCGCCGCGCTCTCCATGAAGGGCGCGCAGGCGCGCACCAACTTCGTCTACGCGCACGCCGCCT

ACAACAACTACCCGCCCTTCCTCGCGCCGTTCCACGCGCAGCACCAGCCCGCCGCCTACGCCGCGTCCTCGGCCATG
CCGTACGGCGGCCAGCAGCAGCACGCGGGCGCGGGGCCGCCGCACATTGGCAGCTCGTACCACCACGGCCACGGCTA
CCACCAGCAGGGCCCGGGCGAGTGCTCCATGCCGGTGCCCAGTGCCGCGGATCACGGCGCCAGCGGCCCGATGGACG
TGCGCGGCAGCAGCGGCCACGACTTCCTCTTCCCCAGCGCCGACGACAACTCCGGGTACCTGAGCAGCGTGGTGCCG
GAGAGCTGCCTCCGGCCCCGCGGCGGCGACCTGCAGGACGCGCGGCGCTACTCCGTGTCCGACGCCGACGCCTACGG
GCTGGGCCTCCGGGAGGACGTGGACGACCTGGCGACGATGGTGGCCGGCTTCTGGGGCGGCGCCGACGCGCCGTACG
GCGGCGGCCACGACATGGTCGCCTCGTCGCAGGGCTCGGACAACGGCTACTCCCCCTTCAGCTTCCTCTCCCACTGA
aacggacgcgctggcattgctcttagcctcttacgtactcagtggtctgctgtgcccaccgtcggccattagctagg
tactgtttgcttcgtgagttcgatcgatcgatcatagtagaagatcgtgaagaagagtggctttgttgtttcggtgc
cctagcttagcggatgctcacatccatgtacttaggttctttcaattagcagcactattactacgtactactagtac
tagtaagttttcatgcatgcacgagtgacatgcttaagttatctctctctaaaattattacagcctctctctaatta
gttagtcgtgtggaattaaagtggcggatcgagagtactcgagaggcccgggaatgtagtacgcacacgcacactgt
tgttgatccttgaaagcttcagttttgggttgtagttttgtttgattcagataggccggccggaggaacatgtcaaa
gtacgtactctcctacgtacttacctcaaggaagaagataaggggaatagtacacggaccacgtactagacgtagta
ctacccattgacagttcaacggaaaggtagatgttcaggacaagtgtcaagcaggcgacctcctttttttttcttgg
gtcgccaggtacttggtgctgattctagggtacgtcacgctcagagcctcagacccatccatcagatgatcagaggg
ccatgcacaaaatgtatcctcttctatgctggtgcagggcttatatataaaccctttcatttcctccgtagcttgct
tggatttgttggatgggaaatgttcttattaatcaatagcaaaaaaaggagaagatataaatgtgcatgtaaccatt
tgagtttattggacaatcaaacaaattcagttttgccagatcgctatatagtgcttgacttgtacaatatttcttat
gacataaatgagacatgtattaccatgcacaaaaagtatatcacacaaaagaacaagcaattgggttcaagtcctaa
ccttggcacggatgctcgcac

>*T. sinskajae* PI 418587

acttactcgtatgttcgtgtcgagaacatcaccaacatatgttggtgaagttattgcggttaatgttttagggaaga
caaaggggctttcattctagtttgcacgtattgcgcgagcaggcacatggacacataagaaagcaaagccatatggt
cagagtgtaaaaaagcacataattatgtagcattttggcatgtatcataatagctagtgtagtactagtatcttgga
gcatagattgcaattgtctctttcaaaacaaatagattacaattcttcaaaaggcttgatgcataggtaggtaagct
ctcttctttcccttcttttcttgtcagtggcaggcatctttacaccatcttattagtttgcaatacttggtactcag
tagtaaatcatcaaagcatgcaaggcagtatgcccaactaacatagatgccacaacaagctatgcatgtaggcccat
taataattattaaagtgtttgtcttggcaggggcttcaatcggctctggcttcagaaccagtgtgtcatatactcat
atgctggttgattaatttggagtgggctagaatcatgacaaagataatcatatctgaaaacgacactaattaatact
tatttgcaaggattgtggcatgcataatattcataaatgaattgtgtgcatgaaaactaagagtagaacattcttac
ttccaagtgttatttttctagcaacacatacacacacanatatatagctatatgtttgactggcacaacattctcct
catctctttggtcctaagattagggcacacaaatccaaacacaaggtgcatacatacaaacttgcagtgagctttcc
attccattccctcctttttactcctagaattaaacaagtgcagcgcatgcatgtccctcccctcctcccctacctcgc
catctcatccacagtgctctcactccttctagcttctccctcctccgagctccccatttaaataccaccctgcctcc
ctccatagtgagcactacacactagaagctcacagtctcagcaaccacctcctcaacctaagctagctagctcacac
ctcagagctcaagctaggcgggagcagtagtatagtagccagccaacctcacttcacttctgccATGAGCTCTCGCA
GCAGCAGCGGCGGCGGCGGTGCCTCCCAGATGATGGCCTTCTCGGAGCATTCGCTGCCGAAGCCGATCGCCGGTCAC
CCGCAGCCGCAGCCGTCCCCGCCGTCGTCGCCGAGCGAGCGGCCGGCGGCGCGCGGCAGGCGGCGCGCGCAGGAGCC
CGGGCGCTTCCTGGGCGTGCGCCGGCGGCCGTGGGGCCGGTACGCGGCCGAGATACGCGACCCGACCACCAAGGAGC
GGCACTGGCTCGGCACATTCGACACGGCGCAGGAGGCCGCCCTGGCCTACGACCGCGCCGCGCTCTCCATGAAGGGC
GCGCAGGCGCGCACCAACTTCGTCTACGCGCACGCCGCCTACAACAACTACCCGCCCTTCCTCGCGCCGTTCCACGC

GCAGCACCAGCCCGCCGCCTACGCCGCGTCCTCGGCCATGCCGTACGGCGGCCAGCAGCAGCACGCGGGCGCGGGGC
CGCCGCACATTGGCAGCTCGTACCACCACGGCCACGGCTACCACCAGCAGGGCCCGGGCGAGTGCTCCATGCCGGTG
CCCAGTGCCGCGGATCACGGCGCCAGCGGCCCGATGGACGTGCGCGGCAGCAGCGGCCACGACTTCCTCTTCCCCAG
CGCCGACGACAACTCCGGGTACCTGAGCAGCGTGGTGCCGGAGAGCTGCCTCCGGCCCCGCGGCGGCGACCTGCAGG
ACGCGCGGCGCTACTCCGTGTCCGACGCCGACGCCTACGGGCTGGGCCTCCGGGAGGACGTGGACGACCTGGCGACG
ATGGTGGCCGGCTTCTGGGGCGGCGCCGACGCGCCGTACGGCGGCGGCCACGACATGGTCGCCTCGTCGCAGGGCTC
GGACAACGGCTACTCCCCCTTCAGCTTCCTCTCCCACTGAaacggacgcgctggcattgctcttagcctcttacgta
ctcagtggtctgctgtgcccaccgtcggccattagctaggtactgtttg

>*T. durum* LD222

gcaggcatctttacaccatcttattagtttgcaatacttggtactcagtagtaaatcatcaaagcatgcaaggcagt
atgcccaactaacatagatgccacaacaagctatgcatgtaggcccattaataattattaaagtgtttgtcttggca
ggggcttcaatcggctctggcttcagaaccagtgtgtcatatactcatatgctggttgattaatttggagtgggcta
gaatcatgacaaagataatcatatctgaaaacgacactaattaatacttatttgcaaggattgtggcatgcataata
ttcataaatgaattgtgtgcatgaaaactaagagtagaacattcttacttccaagtgttatttttctagcaacacat
acacacacacatatatagctatatgtttgactggcacaacattctcctcatctctttggtcctaagattagggcaca
caaatccaaacacaaggtgcatacatacaaacttgcagtgagctttccattccattccctcctttttactcctagaat
taaacaagtgcagcgcatgcatgtccctcccctcctcccctacctcgccatctcatccacagtgctctcactccttc
tagcttctccctcctccgagctccccatttaaataccaccctgcctccctccatagtgagcactacacactagaagc
tcacagtctcagcaaccacctcctcaacctaagctagctagctcacacctcagagctcaagctaggcgggagcagta
gtatagtagccagccaacctcacttcacttctgccATGAGCTCTCGCAGCAGCAGCGGCGGCGGCGGTGCCTCCCAG
ATGATGGCCTTCTCGGAGCATTCGCTGCCGAAGCCGATCGCCGGTCACCCGCAGCCGCAGCCGTCCCCGCCGTCGTC
GCCGAGCGAGCGGCCGGCGGCGCGCGGCAGGCGGCGCGCGCAGGAGCCCGGGCGCTTCCTGGGCGTGCGCCGGCGGC
CGTGGGGCCGGTACGCGGCCGAGATACGCGACCCGACCACCAAGGAGCGGCACTGGCTCGGCACCTTCGACACGGCG
CAGGAGGCCGCCCTGGCCTACGACCGCGCCGCGCTCTCCATGAAGGGCGCGCAGGCGCGCACCAACTTCGTCTACGC
GCACGCCGCCTACAACAACTACCCGCCCTTCCTCGCGCCGTTCCACGCGCAGCACCAGCCCGCCGCCTACGCCGCGT
CCTCGGCCATGCCGTACGGCGGCCAGCAGCAGCACGCGGGCGCGGGGCCGCCGCACATTGGCAGCTCGTACCACCAC
GGCCACGGCTACCACCAGCAGGGCCCGGGCGAGTGTTCCATGCCGGTGCCCAGTGCCGCGGATCACGGCGCCAGCGG
CCCGATGGACGTGCGCGGCAGCAGCGGCCACGACTTCCTCTTCCCCAGCGCCGACGACAACTCCGGGTACCTGAGCA
GCGTGGTGCCGGAGAGCTGCCTCCGGCCCCGCGGCGGCGACCTGCAGGACGCGCGGCGCTACTCCGTGTCCGACGCC
GACGCCTACGGGCTGGGCCTCCGGGAGGACGTGGACGACCTGGCGACGATGGTGGCCGGCTTCTGGGGCGGCGCCGA
CGCGCCGTACGGCGGCGGCCACGACATGGTCGCCTCGTCGCAGGGCTCGGACAACGGCTACTCCCCCTTCAGCTTCC
TCTCCCACTGAaacggacgcgctggcattgctcttagcctcttacgtactcagtggtctgctgtgcccaccgtcggc
cattagctaggtagtgtttgcttcgtgagttcgatcgatcgatcatagtagaagatcgtgaagaagagtggctttgt
tgtttcggtgccctagcttagcggatgctcacatccatgtacttaggttctttcaattagcagcactattactacgt
actactagtactagtaagttttcatgcatgcacgagtgacatgcttaagttatctctctctaaaattattacagcct
ctctctaattagttagtcgtgtggaattaaagtggcggatcgagagtactcgagaggcccgggaatgtagtacgcac
acgcacactgttgttgatccttgaaagcttcagttttgggttgtagttttgtttgattcagataggccggccggagg
aacatgtcaaagtacgtactctcctacgtacttacctcaaggaagaagataagggaaatagtacacggaccacgtac
tagacgtagtactacccattgacagttcaacggaaaggtagatgttcaggacaagtgtcaagcaggcgacctccttt
tttttcttgggtcgccactcgccaggtacttggtgctgattctagggtacgtcacgctcagagcctcagacccattc
atcagatgatcagagggccatgcacaaaatgtatcctcttctatgctggtgcagggcttatatataaaccctttcat
ttcctccgtagcttgcttggatttgttggatgggaaatgttcttattaatcaatagcaaaaaaaggagaagatataa

atgtgcatgtaaccatttgagtttattggacaatcaaacaaattcagttttgccagatcgctatatagtgcttgact
tgtacaatatttcttatgacataaatgagacatgtattaccatgcacaaaaagtatatcacacaaaagaacaagcaa
ttgggttcaagtcctaaccttggcacggatgctcgcactttgctagattttttaaggaacattttgctagatttat
tcaagacattccgacgatatttattcagtaggagagatgttcacatcgacagagaggtgcgttggttctccgttgcg
tcagatatgggggggttcccctttctcctgctgcctctgtcgtcgttccggtggtttgcggctgcaggctccggtggt
ggtgagccctggtggtgtactttgggtctgggggggaaccctggccagtctggccggcccggcggcggcgatgcccat
gggcgccgc

>*T. turgidum* K-40750

atacttggtactcagtagtaaatcatcaaagcatgcaaggcagtatgcccaactaacatagatgccacaacaagcta
tgcatgtaggcccattaataattattaaagtgtttgtcttggcaggggcttcaatcggctctggcttcagaaccagt
gtgtcatatactcatatgctggttgattaatttggagtgggctagaatcatgacaaagataatcatatctgaaaacg
acactaattaatacttatttgcaaggattgtggcatgcataatattcataaatgaattgtgtgcatgaaaactaaga
gtagaacattcttacttccaagtgttatttttctagcaacacatacacacacacatatatagctatatgtttgactg
gcacaacattctcctcatctctttggtcctaagattagggcacacaaatccaaacacaaggtgcatacatacaaact
tgcagtgagctttccattccattccctcctttactcctagaattaaacaagtgcagcgcatgcatgtccctcccct
cctcccctacctcgccatctcatccacagtgctctcactccttctagcttctccctcctccgagctccccatttaaa
taccaccctgcctccctccatagtgagcactacacactagaagctcacagtctcagcaaccacctcctcaacctaag
ctagctagctcacacctcagagctcaagctaggcgggagcagtagtatagtagccagccaacctcacttcacttctg
ccATGAGCTCTCGCAGCAGCAGCGGCGGCGGCGGTGCCTCCCAGATGATGGCCTTCTCGGAGCATTCGCTGCCGAAG
CCGATCGCCGGTCACCCGCAGCCGCAGCCGTCCCCGCCGTCGTCGCCGAGCGAGCGGCCGGCGGCGCGCGGCAGGCG
GCGCGCGCAGGAGCCCGGGCGCTTCCTGGGCGTGCGCCGGCGGCCGTGGGGCCGGTACGCGGCCGAGATACGCGACC
CGACCACCAAGGAGCGGCACTGGCTCGGCACCTTCGACACGGCGCAGGAGGCCGCCCCGGCCTACGACCGCGCCGCG
CTCTCCATGAAGGGCGCGCAGGCGCGCACCAACTTCGTCTACGCGCACGCCGCCTACAACAACTACCCGCCCTTCCT
CGCGCCGTTCCACGCGCAGCACCAGCCCGCCGCCTACGCCGCGTCCTCGGCCATGCCGTACGGCGGCCAGCAGCAGC
ACGCGGGCGCGGGGCCGCCGCACATTGGCAGCTCGTACCACCACGGCCACGGCTACCACCAGCAGGGCCCGGGCGAG
TGTTCCATGCCGGTGCCCAGTGCCGCGGATCACGGCGCCAGCGGCCCGATGGACGTGCGCGGCAGCAGCGGCCACGA
CTTCCTCTTCCCCAGCGCCGACGACAACTCCGGGTACCTGAGCAGCGTGGTGCCGGAGAGCTGCCTCCGGCCCCGCG
GCGGCGACCTGCAGGACGCGCGGCGCTACTCCGTGTCCGACGCCGACGCCTACGGGCTGGGCCTCCGGGAGGACGTG
GACGACCTGGCGACGATGGTGGCCGGCTTCTGGGGCGGCGCCGACGCGCCGTACGGCGGCGGCCACGACATGGTCGC
CTCGTCGCAGGGCTCGGACAACGGCTACTCCCCCTTCAGCTTCCTCTCCCACTGAaacggacgcgctggcattgctc
ttagcctcttacgtactcagtggtctgctgtgcccaccgtcggccattagc

>*T. durum* R107

tacttactcgtatgttcgtgtcgagaacatcaccaacatatgttggtgaagttattgcggttaatgttttagggaag
acaaaaggggctttcattctagtttgcacgtattgcgcgagcaggcacatggacacataagaaagcaaagccatatg
gtcagagtgtaaaaaagcacataattatgtagcattttggcatgtatcataatagctagtgtagtactagtatcttg
gagcatagattgcaattgtctctttcaaaacaaatagattacaattcttcaaaaggcttgatgcataggtaggtaag
ctctcttctttcccttcttttcttgtcagtggcaggcatctttacaccatcttattagtttgcaatacttggtactc
agtagtaaatcatcaaagcatgcaaggcagtatgcccaactaacatagatgccacaacaagctatgcatgtaggccc
attaataattattaaagtgtttgtcttggcaggggcttcaatcggctctggcttcagaaccagtgtgtcatatactc
atatgctggttgattaatttggagtgggctagaatcatgacaaagataatcatatctgaaaacgacactaattaata
cttatttgcaaggattgtggcatgcataatattcataaatgaattgtgtgcatgaaaactaagagtagaacattctt
acttccaagtgttatttttctagcaacacatacacacacacatatatagctatatgtttgactggcacaacattctc

ctcatctctttggtcctaagattagggcacacaaatccaaacacaaggtgcatacatacaaacttgcagtgagcttt
ccattccattccctccttttactcctagaattaaacaagtgcagcgcatgcatgtccctcccctcctcccctacctc
gccatctcatccacagtgctctcactccttctagcttctccctcctccgagctccccatttaaataccaccctgcct
ccctccatagtgagcactacacactagaagctcacagtctcagcaaccacctcctcaacctaagctagctagctcac
acctcagagctcaagctaggcgggagcagtagtatagtagccagccaacctcacttcacttctgccATGAGCTCTCG
CAGCAGCAGCGGCGGCGGCGGTGCCTCCCAGATGATGGCCTTCTCGGAGCATTCGCTGCCGAAGCCGATCGCCGGTC
ACCCGCAGCCGCAGCCGTCCCCGCCGTCGTCGCCGAGCGAGCGGCCGGCGGCGCGCGGCAGGCGGCGCGCGCAGGAG
CCCGGGCGCTTCCTGGGCGTGCGCCGGCGGCCGTGGGGCCGGTACGCGGCCGAGATACGCGACCCGACCACCAAGGA
GCGGCACTGGCTCGGCACCTTCGACACGGCGCAGGAGGCCGCCCCGGCCTACGACCGCGCCGCGCTCTCCATGAAGG
GCGCGCAGGCGCGCACCAACTTCGTCTACGCGCACGCCGCCTACAACAACTACCCGCCCTTCCTCGCGCCGTTCCAC
GCGCAGCACCAGCCCGCCGCCTACGCCGCGTCCTCGGCCATGCCGTACGGCGGCCAGCAGCAGCACGCGGGCGCGGG
GCCGCCGCACATTGGCAGCTCGTACCACCACGGCCACGGCTACCACCAGCAGGGCCCGGGCGAGTGTTCCATGCCGG
TGCCCAGTGCCGCGGATCACGGCGCCAGCGGCCCGATGGACGTGCGCGGCAGCAGCGGCCACGACTTCCTCTTCCCC
AGCGCCGACGACAACTCCGGGTACCTGAGCAGCGTGGTGCCGGAGAGCTGCCTCCGGCCCCGCGGCGGCGACCTGCA
GGACGCGCGGCGCTACTCCGTGTCCGACGCCGACGCCTACGGGCTGGGCCTCCGGGAGGACGTGGACGACCTGGCGA
CGATGGTGGCCGGCTTCTGGGGCGGCGCCGACGCGCCGTACGGCGGCGGCCACGACATGGTCGCCTCGTCGCAGGGC
TCGGACAACGGCTACTCCCCCTTCAGCTTCCTCTCCCACTGAaacggacgcgctggcattgctcttagcctcttacg
tactcagtggtctgctgtgcccaccgtcggccattagctaggtagtgtttgcttcgtgagttcgatcgatcgatcat
agtagaagatcgtgaagaagagtggctttgttgtttcggtgccctagcttagcggatgctcacatccatgtacttag
gttctttcaattagcagcactattactacgtactactagtactagtaagttttcatgcatgcacgagtgacatgctt
aagttatctctctctaaaattattacagcctctctctaattagttagtcgtgtggaattaaagtggcggatcgagag
tactcgagaggcccgggaatgtagtacgcacacgcacactgttgttgatccttgaaagcttcagttttgggttgtag
ttttgtttgattcagataggccggccggaggaacatgtcaaagtacgtactctcctacgtacttacctcaaggaaga
agataagggaaatagtacacggaccacgtactagacgtagtactacccattgacagttcaacggaaaggtagatgtt
caggacaagtgtcaagcaggcgacctcctttttttttcttgggtcgccactcgccaggtacttggtgctgattctagg
gtacgtcacgctcagagcctcagacccattcatcagatgatcagagggccatgcacaaaatgtatcctcttctatgc
tggtgcagggcttatatataaaccctttcatttcctccgtagcttgcttggatttgttggatgggaaatgttcttat
taatcaatagcaaaaaaaggagaagatataaatgtgcatgtaaccatttgagtttattggacaatcaaacaaattca
gttttgccagatcgctatatagtgcttgacttgtacaatatttcttatgacataaatgagacatgtattaccatgca
caaaaagtatatcacacaaaagaacaagcaattgggttcaagtcctaaccttggcacggatgctcgcactttgctag
atttttttaaggaacattttgctagatttattcaagacattccgacgatatttattcagtaggagagatgttcacat
cgacagagaggtgcgttggttctccgttgcgtcagatatgggggttcccctttctcctgctgcctctgtcgtcgtt
ccggtggtttgcggctgcaggctccggtggtggtgagccctggtggtgtactttgggtttgggggaaccctggcca
gtctggccggcccggcggcggcgatgcccatgggcgccgcttcat
