# Supplementary material for: Genes WHEAT FRIZZY PANICLE and SHAM RAMIFICATION 2 independently regulate differentiation of floral meristems in wheat
Source: BMC Plant Biol. 2017 Dec 28;17(Suppl 2):252. doi: 10.1186/s12870-017-1191-3 (PMC5751757; doi:10.1186/s12870-017-1191-3)

Supplemental Figure S1. Spike with extended spikelet rachilla containing numerous florets (sham ramification). esr - extended spikelet rachilla.

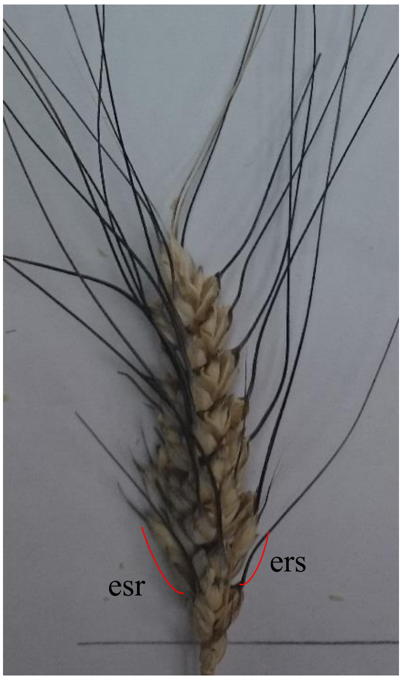

Supplement: Supplementary file 2 — Spike with extended spikelet rachilla containing numerous florets. (PDF 170 kb) [file 12870_2017_1191_MOESM2_ESM.pdf]
